# Ecological Synthesis of Precious Metal Nanoparticles: Harnessing the Potential of Marine Algae Biomass

**DOI:** 10.3390/nano15191492

**Published:** 2025-09-30

**Authors:** Laura Bulgariu

**Affiliations:** Department of Environmental Engineering and Management, “Cristofor Simionescu” Faculty of Chemical Engineering and Environmental Protection, Gheorghe Asachi Technical University of Iaşi, 700050 Iaşi, Romania; laura.bulgariu@academic.tuiasi.ro

**Keywords:** marine algae biomass, precious metal nanoparticles, synthesis efficiency, green method

## Abstract

The synthesis of precious metal nanoparticles (PM-NPs) is an important field of research that has expanded significantly in recent decades due to their numerous applications. Therefore, research has been directed toward developing green methods for the synthesis of such nanoparticles that are simple, safe, eco-friendly, efficient, and sustainable. In this context, the use of marine algae biomass for the green synthesis of PM-NPs can be a viable large-scale alternative, as algae are easy to cultivate, have a rapid growth rate, and are widely distributed across many regions of the globe. The reduction of precious metal ions takes place at the surface of algae biomass particles, and the characteristics of the resulting precious metal nanoparticles depend on the experimental conditions (pH, amount of algae biomass, contact time, etc.), as well as on the type of algae biomass and the speciation form of the metal ions in the solution. All these factors significantly influence the properties of precious metal nanoparticles, and their understanding allows the development of synthesis strategies that can be applied on a large scale. The aim of this review is to provide a comprehensive overview of the way in which PM-NPs can be synthesized using algae biomass. The importance of experimental conditions (such as pH, contact time, amount of biomass, type of algal biomass, temperature, etc.) on the synthesis efficiency, as well as the elementary steps involved in the synthesis, is also discussed in this study. Particular attention has been paid to the analytical methods used for characterizing PM-NPs, as they provide crucial data regarding their structure and composition. These aspects are essential for identifying the practical applications of PM-NPs.

## 1. Introduction

The numerous applications of metallic nanoparticles in various fields, from industrial processes to human health and environmental protection, have led to a significant increase in research in this area over the past decades [1,2]. Therefore, the development of new methods for the synthesis of metallic nanoparticles, as well as new applications for them in key areas relevant to modern societies, remains an open and challenging research direction. Precious metal nanoparticles (PM-NPs, such as Au-NPs, Ag-NPs, Pt-NPs, Pd-NPs) hold a special place in this field [3]. Due to their remarkable physical, chemical, and optoelectronic properties, they serve as the starting point for numerous industrial activities, including materials science, electronics, pharmaceuticals, and the food industry [3,4,5,6], as well as for environmental engineering [7,8,9]. In many of these industrial activities, the use of PM-NPs has successfully replaced the traditional processes, or even allowed the design of new processes that are significantly more efficient from technological, economic, and environmental perspectives [10,11,12]. Under these conditions, the demand for precious metal nanoparticles (PM-NPs) on the market is expected to grow steadily in the future, and this trend is clearly highlighted in many studies from the literature [12,13,14,15]. Therefore, the design of synthesis methods for PM-NPs that are both efficient and environmentally friendly, and that involve the use of low-cost raw materials (or even waste products), is a key factor for the continued development of large-scale applications of these nanomaterials.

Currently, numerous chemical and physical methods (e.g., chemical vapor deposition, sol–gel processes, spinning, pyrolysis, etc.) are reported in the literature for the synthesis of PM-NPs, which aim to efficiently obtain reliable and feasible nanoparticles for industrial applications [16,17,18,19,20]. Unfortunately, most of these synthesis methods present a number of major disadvantages, such as high costs, significant consumption of chemical reagents and/or energy, and considerable toxic potential, which limit their wide-scale applicability. Nevertheless, such methods are used for obtaining metallic nanoparticles that enable the production of materials with special properties, which have applications in the electronics, telecommunications, and space industries, among others [20,21,22,23,24].

Compared to physical or chemical synthesis methods, the use of biological materials for obtaining PM-NPs is much more suitable from an environmental protection perspective. Such biological materials, including microorganisms (e.g., bacteria, viruses, fungi, etc.) [25,26], aquatic plants (e.g., micro- and macroalgae) [27,28], or terrestrial plants (leaves, seeds, stems from various cultivated or perennial plants) [29,30], have proven effective in the synthesis processes of PM-NPs and, moreover, environmentally friendly, as they do not involve the use of toxic chemical reagents. Therefore, synthesis methods of PM-NPs that involve the use of such biological materials can be classified as green and sustainable synthesis methods, which have attracted significant attention from researchers. 

In selecting a suitable biological material for the synthesis of PM-NPs, besides the efficiency of the synthesis process, other aspects must be taken into account. Among these, the most important are the availability of the biological material, the cost of its preparation/use, and any already-established applications of the respective material [27,30]. Thus, although microorganisms (bacteria, viruses, fungi) have demonstrated high efficiency in obtaining PM-NPs, their use requires very well-defined experimental conditions, which may vary within narrow ranges, to ensure their viability [31,32,33]. Moreover, the spaces in which microorganisms are used must be adequately equipped to prevent contamination and risks to the health of people working in such environments. All these precautions involve considerable costs, and therefore, their large-scale use is quite limited. Terrestrial plant residues (leaves, stems, peels, etc.), although easy to obtain in large quantities and at low costs, have moderate efficiency in the synthesis of PM-NPs, and therefore, large amounts are required [34,35,36]. In addition, most of these materials already have established uses (in animal husbandry, the food and cosmetics industries, the pharmaceutical industry, etc.) [37,38], which have proven their economic efficiency.

From this perspective, marine algae represent a much better choice, which is why this type of biological material has received increased attention in recent years. Their availability in large quantities in many regions of the world, low preparation costs, rapid growth rate allowing for multiple harvests, cultivation that does not require agricultural land [39,40,41], and, last but not least, high efficiency in PM-NP synthesis [41,42] are some of the most important advantages that recommend the use of marine algae for this purpose.

However, the term “marine algae” is a general one, which includes all types of micro- and macroalgae, brown (*Phaeophyceae* sp.), red (*Rhodophyceae* sp.), and green (*Chlorophyceae* sp.), that grow in aquatic environments with high salinity, such as the waters of seas or oceans [42,43]. Unfortunately, the potential for using marine algae in the synthesis of PM-NPs varies from one class to another, especially in terms of the implementation of large-scale processes. Thus, although microalgae are recognized for their high yield in obtaining PM-NPs [44,45], they require strict cultivation conditions (nutrients, temperature, light, air flow, etc.) that must be maintained over a fairly long period [46]. Moreover, the separation of microalgae from the obtained PM-NPs is quite difficult due to their comparable sizes [45]. Unlike microalgae, macroalgae can be easily separated from the synthesized PM-NPs through simple and low-cost filtration and washing operations. 

Macroalgae can be used both in live and dried form in PM-NP synthesis processes. However, in the case of live macroalgae, their relatively low efficiency and the need to maintain well-defined cultivation conditions throughout the entire PM-NP synthesis process (which can last several tens of hours) limit their large-scale use [47,48,49]. In the case of dried algae (or algae biomass), most of these drawbacks are minimized. Marine algae are harvested directly from the sea/ocean waters, washed several times (to remove solid impurities and salts from the leaves), and then dried, most often in air at room temperature [50,51]. All these preparation operations are carried out with minimal costs, and the obtained algae biomass, stored under low humidity conditions, can be used over long periods of time (up to several years). Therefore, the use of algal biomass for the synthesis of PM-NPs is much more suitable for industrial-scale applications, and this is the reason why recent research has been increasingly focused in this direction. 

This study aims to provide a comprehensive overview of the potential use of dried algae biomass for the synthesis of PM-NPs and to highlight the potential applications on a large scale. The experimental parameters that influence the efficiency of PM-NP synthesis and that may contribute to increasing the synthesis yield, as well as the mechanisms involved in the synthesis of PM-NPs using algae biomass, are also discussed. Thus, the potential of green chemistry methods in the ecological synthesis of PM-NPs will be highlighted, and the importance of using sustainable materials in these synthesis processes, such as marine algae biomass, will be emphasized.

## 2. Precious Metal Nanoparticles: Characteristics and Applications

In the category of precious metals, the chemical elements included are gold (Au), silver (Ag), platinum (Pt), and palladium (Pd), as well as rhodium (Rh), iridium (Ir), ruthenium (Ru), and osmium (Os) [52]. Among these, only the first four chemical elements (Au, Ag, Pt, Pd) are used in large-scale applications, and therefore, their nanoparticles were considered in this study and generically referred to as precious metal nanoparticles (PM-NPs). 

Precious metal nanoparticles (PM-NPs) exhibit a number of unique characteristics that make them extremely valuable in research and technological applications. These characteristics are the results of their nanometric size and physical and chemical properties, and the most important are:

***(1) Physico-chemical characteristics***:

(i) *High specific surface area*: Due to their nanometric size (between 1 and 100 nm), PM-NPs have a very high surface/volume ratio. This extended surface area gives them increased reactivity and the ability to interact with a large number of molecules in aqueous solution [52,53].

(ii) *Varied geometric shapes*: Depending on the nature of the metal and the synthesis method, PM-NPs can have various geometric shapes (spherical, cubic, star, rod, etc.). The type of geometric shape has an important effect on the optical and chemical properties of PM-NPs [54]. 

(iii) *Surface Plasmon resonance*: This remarkable property is due to the free electrons on the surface of PM-NPs, which can collectively oscillate under the influence of incident light and create a plasmonic resonance. This resonance leads to string absorption and scattering of light at certain wavelengths, determining intense and specific colors for each precious metal [54,55]. 

(iv) *Special optic properties*: The optical properties of PM-NPs are extremely sensitive to size, shape, aggregation, and environment. This makes them ideal for applications in optical sensors. In addition, some PM-NPs emit radiation when excited, a feature particularly useful in optoelectronics and biology [56,57]. 

(v) *Electrical properties*: PM-NPs have good electrical conductivity, a characteristic that is widely used for the development of electronic components and sensor devices [58].

***(2) Biological and biochemical characteristics***: 

(i) *Biocompatibility*: The surface of PM-NPs can be easily modified (functionalized) with a wide range of chemical compounds or biomolecules (proteins, DNA, RNA, lipids, carbohydrates, etc.). This bio-functionalization capability allows them to recognize specific target molecules or participate in complex biological reactions, making them highly attractive for biomedical applications [59,60,61]. 

(ii) *Cellular penetration*: Due to their small size, PM-NPs easily penetrate inside cells and organelles, giving them a high therapeutic or toxicological potential [62]. 

(iii) *Antimicrobial properties (especially silver)*: Silver nanoparticles are known for their strong antimicrobial properties against a wide range of microorganisms, including bacteria, fungi, and viruses. This characteristic makes them useful in medical, cosmetic, and industrial applications [63,64]. 

***(3) Technological characteristics***: 

(i) *Catalysts*: Most PM-NPs are highly efficient catalysts in industrial chemical reactions [65]. 

(ii) *Sensors and Biosensors*: Due to their optical properties and high functionalization capacity, PM-NPs can be used for the detection and quantitative determination of a wide range of chemical compounds (organic or inorganic) in small volumes of samples with complex compositions [66,67]. 

(iii) *Surface-enhanced Raman scattering (SERS)*: PM-NPs have the ability to amplify the Raman signal, which makes them particularly useful for highly sensitive molecular detection [68]. 

**Figure 1:**
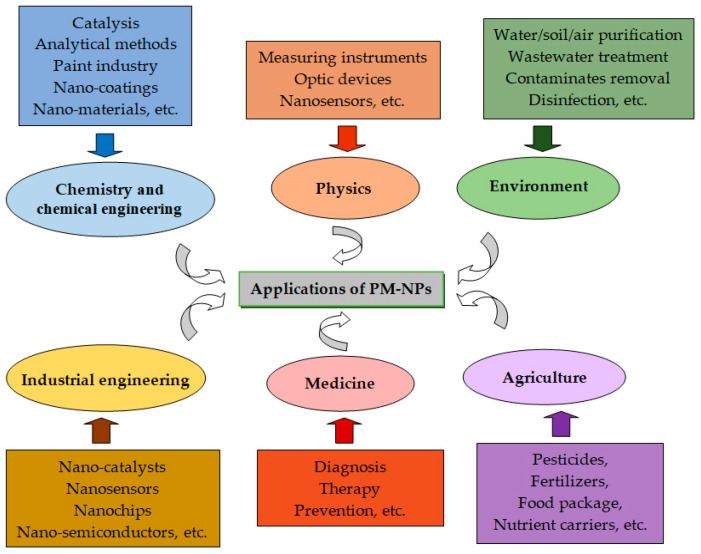
The most important large-scale applications of PM-NPs.

Due to all these characteristics, PM-NPs have become powerful tools over the past decades for the development of new advanced technologies, which have found applications in various fields essential to modern societies (Figure 1). 

However, the large-scale use of PM-NPs also presents several drawbacks. For example, the aggregation of PM-NPs (their tendency to form clusters of nanoparticles) reduces their stability and effectiveness in physical, chemical, and industrial processes [60,61]. Oxidation, particularly in the case of Ag-NPs, is responsible for a significant decrease in antimicrobial and functionalization properties [64]. Last but not least, the toxicity of PM-NPs must be rigorously evaluated to prevent the deterioration of environmental quality and human health [52,54]. All of these drawbacks can be significantly reduced by choosing an appropriate synthesis process for PM-NPs, and these considerations formed the basis for selecting methods that use biological materials over physical and chemical synthesis methods.

## 3. Synthesis Mechanism of PM-NPs Using Algae Biomass

Except for physical synthesis methods, which involve the disruption of precious metal particle aggregates down to the nanoparticle level, the synthesis mechanism of PM-NPs is based mainly on a redox process that involves the reduction of precious metal ions, generally from aqueous media, to the zero oxidation state, according to the redox process:

PM^n+^ + n e^−^ → PM^0^ (1)

where PM is the precious metal element (Au, Ag, Pt, Pd), and n is the number of electrons involved in the redox process.

In the case of precious metals, this reduction process occurs spontaneously (they are positioned after hydrogen in the electrochemical activity series), with a high rate, and due to the chemical particularities of these elements (Table 1), the formed PM^0^ species are stable for long periods of time and do not tend to revert to their oxidized form (PM^n+^). That is why they are also called noble metals [69]. 

The electrons required for the reduction processes (Equation (1)) can be provided by a chemical reducing agent (in the case of chemical synthesis methods) or by the constituents of biological cells (in the case of synthesis methods employing biological materials) [17,69]. 

Due to the significant limitations of chemical synthesis methods (as outlined in the previous section), the use of biological materials for the synthesis of PM-NPs offers several advantages, allowing them to be classified among environmentally friendly (green) synthesis methods [70,71]. In the case of using biological materials, the synthesis of PM-NPs takes place under mild conditions (at ambient temperature and pressure), with low energy consumption and in the absence of highly toxic chemical reagents [71,72]. In addition, by using biological materials, some PM-NPs with special properties can be obtained, which are much more difficult to synthesize using traditional physical or chemical methods [17,70]. All of these significant advantages recommend the use of biological materials for the synthesis of PM-NPs and have served as the starting point in designing new green methods for nanoparticle synthesis.

But in order to ensure the sustainability and economic efficiency on a large scale of such PM-NP synthesis methods, the biological material must be selected so that (i) it is easy to obtain and use, (ii) it is available in sufficient quantities and for longer periods of time, and (iii) it does not require special conditions for use that would significantly increase the cost of the synthesis methods. 

All these conditions are met by the dried biomass of marine algae (algae biomass), which is why its use in the synthesis of PM-NPs remains an open and attractive research direction. Moreover, since algae biomass is already dried (and does not require special cultivation conditions), residual solutions or those obtained from recycling processes that contain precious metal ions can also be used for PM-NP synthesis, even if such solutions (wastewater) have low concentrations of precious metal ions and a complex composition (a large number of components) [73]. 

**Figure 2:**
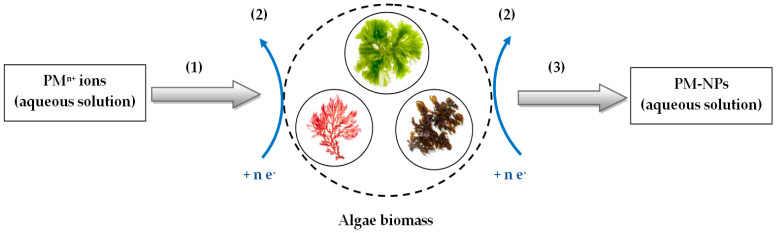
Schematic illustration of the elementary processes involved in the synthesis of PM-NPs using algae biomass.

In general, the synthesis mechanism of PM-NPs using algae biomass involves three successive main steps (Figure 2), namely: 

(1) The transport of precious metal ions to the extracellular surface of algae biomass; 

(2) Reduction of precious metal ions by functional groups on the extracellular surface of algae biomass;

(3) Transport of PM-NPs in the bulk solution and their stabilization. 

***(1) Transport of precious metal ions to the extracellular surface of algae biomass***—this is carried out through elementary diffusion processes, and the driving force is determined by the concentration of precious metal ions in the aqueous solution and by the mobility of these ions [74]. When algae biomass is added to the solution containing precious metal ions, they tend to spontaneously move toward the surface of the biomass particles, due to interface phenomena that occur when the aggregation state changes (liquid (aqueous solution)/solid (algae biomass)) [75]. Thus, the higher the concentration of precious metal ions and their mobility, the more easily the elementary diffusion processes occur, and the more efficient their transport to the surface of the algae biomass becomes. 

In general, the mobility of an ionic species depends on the speciation form and the number of electric charges. The larger the volume of the ionic species and the smaller the number of electric charges, the lower its mobility [69]. This is particularly important from an experimental point of view. If precious metal ions are present in solution in complex form (especially with organic ligands) or in hydrolyzed form (basic salts of these ions), their mobility is significantly reduced. Therefore, it is preferable to use mineral acid solutions (HX) when preparing precious metal ion solutions for PM-NP synthesis, which have the role of (i) ensuring the simplest possible form of speciation (AuX_4_^−^, Ag^+^, PtX_4_^2−^ or PdX_4_^2−^), and thus high mobility; (ii) preventing the precipitation of precious metal ions due to possible hydrolysis processes; and (iii) eliminating or degrading any traces of organic compounds (fats, detergents, etc.) that could block the surface of algae particles. 

Most often, for preparing solutions of precious metal ions, a mixture of HNO_3_ and HCl is recommended [52,62]. The chemical species formed in this case belong to the class of chloride complexes, which can readily participate in reduction processes (Table 2). Silver is an exception. In the case of silver, only HNO_3_ is recommended, as the presence of HCl leads to the formation of AgCl, a stable and poorly soluble compound [52] that is less likely to participate in reduction processes (Table 2). 

***(2) Reduction of precious metal ions by functional groups on the extracellular surface of algae biomass***—responsible for the reduction of precious metal ions in the aqueous solution are the functional groups found on the extracellular surface of the algae biomass. It is well known that the extracellular membrane of algal biomass is composed of a wide variety of organic compounds (such as complex polysaccharides, proteins and glycoproteins, pigments, phenolic compounds, lipids, fatty acids, exopolymers, etc.) [28,76,77]. 

**Figure 3:**
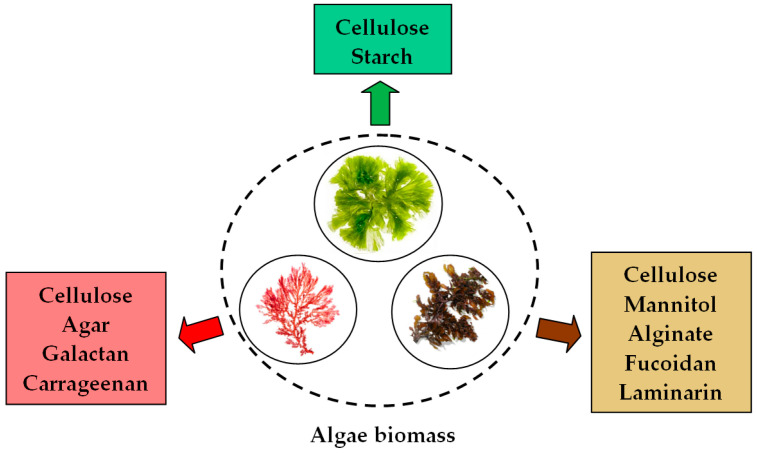
The main components of the extracellular surface of algae biomass.

**Figure 4:**
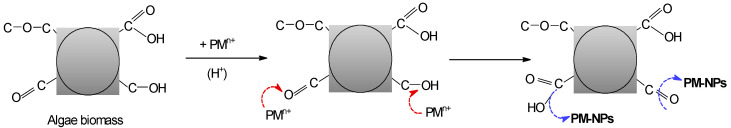
Schematic illustration of the redox processes at the surface of algae biomass.

Although the composition of the extracellular walls varies significantly depending on the type of marine algae (brown, red, or green) (Figure 3) and is influenced by environmental conditions and the developmental stage of the algae prior to harvesting [77], all these constituents contain in their structure hydroxyl (–OH), carbonyl (–C=O), carboxyl (–COOH), ether (–C–O–C–), amino (–NH_2_), phosphate groups, etc., which can provide the electrons necessary for the reduction of precious metal ions [78,79]. Among these functional groups, the most likely to participate in redox processes are the aldehydic carbonyl groups (standard potential = +0.58 V) and the phenolic hydroxyl groups (standard potential = +0.5 to +0.8 V) [52]. Figure 4 illustrates a schematic representation of the redox processes involved in the synthesis of PM-NPs.

The other functional groups, although not directly contributing to the reduction of precious metal ions in aqueous solution (due to their high stability), can indirectly influence the efficiency of redox processes (by modifying the spatial conformation of the compounds that make up the extracellular surface, or the charge density on the donor atom). Therefore, it is expected that the algae that have a high content of sulfated polysaccharides and polyphenolic compounds will have a higher efficiency in the synthesis of PM-NPs and be recommended for such applications. 

This is confirmed by studies in the literature showing that brown and red algae, which are rich in phenolic compounds (especially bromophenols, tannins, and flavonoids), allow for the efficient synthesis of PM-NPs, which takes place in a short period of time [79,80,81,82,83]. Table 3 presents some examples of marine algae biomass successfully used in the synthesis of some PM-NPs. 

**Figure 5:**
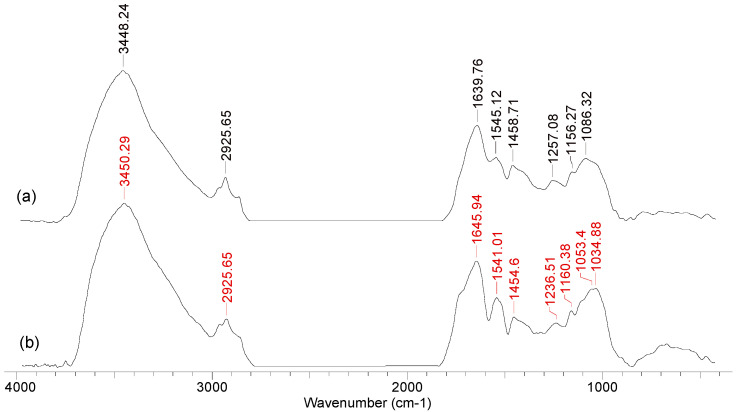
FTIR spectra of *Callithamnion corymbosum* (red algae biomass) before (**a**) and after (**b**) synthesis of Au-NPs.

The structural changes in the functional groups on the surface of the algae biomass after the synthesis of PM-NPs can be easily highlighted using FTIR spectra. For example, Figure 5 illustrates the FTIR spectra recorded for *Callithamnion corymbosum* red algae biomass, before and after the synthesis of Au-NPs.

The most significant shifts of the absorption maxima can be observed in the case of the bands at (Figure 5) (i) 3448 cm^−1^—corresponding to the O–H bond in saturated or aromatic alcohols; (ii) 1639 cm^−1^—assigned to the carbonyl groups in aldehydes conjugated with a double bond or aromatic ring; (iii) 1257 cm^−1^—which is typical for the C–O bond in phenols/aromatic alcohols; and (iv) 1086 cm^−1^—characteristic of C–O–C bonds in oxygenated compounds (saturated or aromatic). 

However, the interpretation of the FTIR spectra in this case must be carried out with increased caution. As can also be seen in Figure 5, the changes in absorption maxima and band intensity for the algae biomass after PM-NP synthesis are not particularly significant (compared to the initial algae biomass). This has two particularly important consequences. On one hand, increased attention must be paid to the preparation of the algae biomass samples (washing, drying, weighing, etc.) that are to be used for the recording of FTIR spectra. On the other hand, after the synthesis of PM-NPs, the algae biomass still has sufficient functional groups to be used in multiple synthesis cycles. This last aspect, although particularly important from a practical point of view, is rarely addressed in the literature and represents one of the challenges that future studies must overcome in order to facilitate the large-scale implementation of such PM-NP synthesis processes.

Since polyphenolic compounds and polysaccharides play an important role in the synthesis of PM-NPs [11,94], one of the proposed solutions to enhance the efficiency of synthesis processes is the extraction of such compounds from algae biomass and the use of the obtained solutions (algae extracts) in the synthesis processes [95]. Most likely, this approach was based on the observation that, regardless of their type (brown, red, or green algae), when dried algae biomass is placed in an aqueous solution, the resulting solution, after filtration, is opalescent or even colored. This change in the appearance of the aqueous solution is due to the solubilization of organic components from the composition of algae biomass (pigments, tannins, polyphenols, polysaccharides, etc.) [94]; that is, precisely those compounds that play an important role in the synthesis of PM-NPs. Under these conditions, the use of algae extracts instead of algae biomass would allow for a more intimate contact between precious metal ions and the organic molecules that provide the electrons required for reduction and would significantly improve the efficiency of PM-NP synthesis. This increase in the efficiency of PM-NP synthesis is also due to the significant reduction in the time required to carry out elementary steps 1 and 3 (see page 6), from approximately one hour when using algae biomass to a maximum of 20 min when using algae extracts [96].

Moreover, in most cases, algae extracts are relatively easy to obtain by mixing dried algae biomass with hot distilled water (40–80 °C), vigorous stirring for several hours (1–6 h) [97], followed optionally by a purification step using polar solvents (most commonly ethanol) [94,96], in order to increase the concentration of organic compounds solubilized from the algae biomass. Table 4 presents several examples of obtaining extracts from algae biomass, with applications in the synthesis of PM-NPs. 

**Figure 6:**
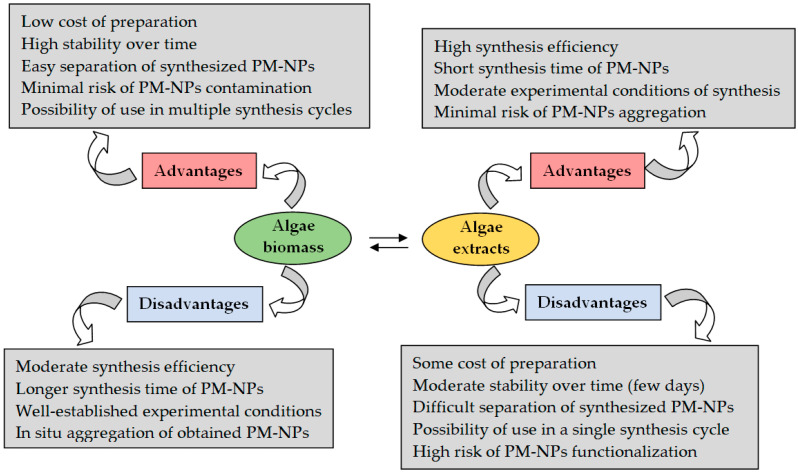
The main advantages and disadvantages of using algae biomass and algae extracts in the synthesis of PM-NPs.

Nevertheless, the use of algae extracts also has a number of drawbacks. Figure 6 illustrates the main advantages and disadvantages of using algae biomass and algae extracts in the synthesis of PM-NPs.

However, the type of algae biomass is not the only factor influencing the efficiency of PM-NP synthesis. The concentration of precious metal ions in the solution, as well as the experimental conditions (pH, biomass amount, ionic strength, contact time, temperature, etc.), can significantly influence the efficiency of PM-NP synthesis [94,96] and, therefore, these factors should also be considered in the selection of the appropriate algae biomass. 

***(3) Transport of PM-NPs in the bulk solution and their stabilization***—once formed, PM-NPs tend to spontaneously detach from the surface of biomass particles and migrate into the aqueous solution. This migration is mainly determined by (i) the low oxidation tendency and stable electronic structure of the formed PM-NPs [11,49] and (ii) the decrease in the reducing properties of the functional groups on the surface of the algae biomass (as a result of their oxidation during the redox processes involved in PM-NP synthesis—see Figure 4) [17,69]. This represents a real advantage in using algae biomass for the synthesis of PM-NPs, as it allows the obtained nanoparticles to be separated relatively easily through simple operations (e.g., filtration).

However, once they reach the bulk of the solution, PM-NPs have relatively low stability and tend to agglomerate. This tendency to agglomerate is well known and is due to the fact that PM-NPs have a large specific surface area and high surface energy, which gives them increased reactivity [52,53].

Because of their high reactivity, PM-NPs can easily engage in physical and/or electrostatic interactions, which favor their agglomeration [53]. This is undesirable, especially when aiming to synthesize PM-NPs with small and controlled dimensions. To prevent the agglomeration of the formed nanoparticles, stabilizing agents are added directly to the aqueous solution used for the synthesis of PM-NPs. 

There are two types of agents that can be mainly used to stabilize the synthesized PM-NPs, namely (1) strong electrolytes and (2) organic molecules (organic macromolecules or organic polymers) [86,107]. Both categories of stabilizing agents serve to reduce the reactivity of PM-NPs in aqueous solution, in accordance with the principles of colloidal chemistry [108]. 

**Figure 7:**
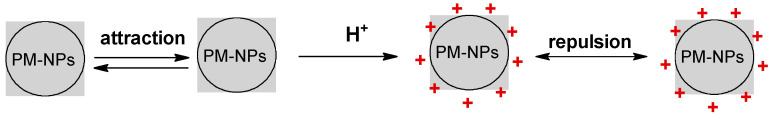
Schematic illustration of PM-NP dispersion in the presence of HNO_3_.

Among strong electrolytes, HNO_3_ solutions of various concentrations (10^−3^–10^−1^ mol/L) are frequently used for this purpose [86]. This is because the presence of HNO_3_ prevents the involvement of precious metal ions in secondary processes (such as hydrolysis, precipitation, etc.), favors their participation in redox (reduction) processes, and, after the synthesis of PM-NPs, creates a favorable environment for dispersion [108]. This media favorable to dispersion is determined by the presence of protons (H^+^), which, due to their small size and high mobility, tend to cover the surface of PM-NPs and form a layer of electric charges that prevents the agglomeration of nanoparticles (Figure 7). 

The use of HNO_3_ as a stabilizing agent is suitable for the synthesis processes of PM-NPs using algae biomass, as it ensures (i) an appropriate pH value for the development of redox (reduction) reactions, (ii) protonation of the functional groups on the surface of the algae biomass without degrading them (due to its weak oxidizing character), and (iii) a high concentration of H^+^ that can prevent the aggregation of the formed nanoparticles. However, the presence of high concentrations of HNO_3_ in the aqueous solution, besides having a moderate dispersion efficiency, increases the risk that, once formed, PM-NPs may dissolve (PM-NPs → PMⁿ^+^), precisely due to the excessively high concentration of protons.

**Figure 8:**
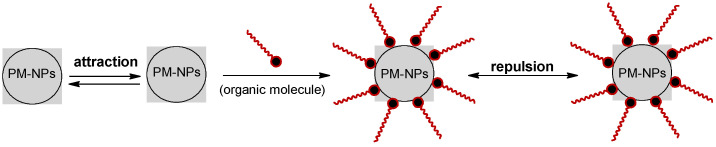
Schematic illustration of PM-NPs dispersion by surface functionalization.

Organic compounds are much more effective stabilizing agents for PM-NPs than HNO_3_. Moreover, they can be used both in the case of algae biomass and algae extracts, allowing the synthesis of PM-NPs with well-defined sizes and shapes that remain stable for acceptable periods of time (several weeks) [109]. The stabilization of PM-NPs in this case can be achieved in two ways: 

(a) ***Through electrostatic repulsion*** (as illustrated in Figure 7), when electrically charged organic molecules are adsorbed onto the PM-NP surface, preventing their agglomeration. 

(b) ***Through functionalization of the PM-NPs surface*** (Figure 8), when organic molecules are bound to the surface of the PM-NPs, forming an “organic” layer that prevents their agglomeration.

Table 5 presents several examples of organic compounds that are used as stabilizing agents for PM-NPs. 

For the selection of organic stabilizing agents in the synthesis processes of PM-NPs, the following aspects must be considered: (i) the organic compounds should be easily soluble in aqueous media—to avoid the need for using organic solvents, which contradicts the principles of green synthesis methods; (ii) they should be stable under the experimental synthesis conditions (acid media, high ionic strength) and exhibit low coagulation tendency—thereby ensuring the stability of the obtained PM-NPs and enhancing the efficiency of the stabilizing agent; and (iii) the binding to the surface of PM-NPs should be reversible, so that the practical applicability of the synthesized nanoparticles is preserved. 

## 4. Experimental Factors Influencing the Synthesis of PM-NPs

**Figure 9:**
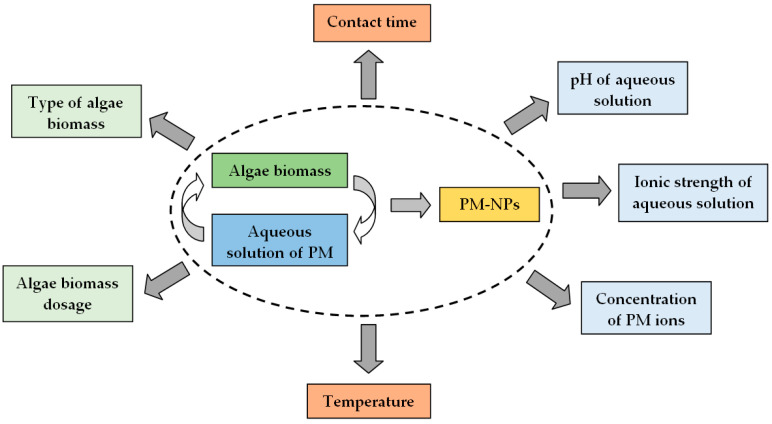
The most important experimental parameters influencing the efficiency of PM-NP synthesis using algae biomass (PM—precious metal ions).

The efficiency of the synthesis processes, as well as the shape and size of the obtained PM-NPs, depends on the efficiency with which the three elementary steps, discussed in detail in the previous section, are carried out. Each of these three steps is influenced by a series of experimental parameters characteristic of the algae biomass or aqueous solution (e.g., type of algae biomass, biomass quantity, pH, ionic strength, concentration of precious metal ions, contact time, temperature, etc.). Selecting appropriate values for each of these parameters can improve the efficiency of PM-NP synthesis processes, potentially making them suitable for large-scale applications. The most important experimental parameters that can significantly influence the efficiency of PM-NP synthesis processes are illustrated in Figure 9 and will be discussed in detail in this section. 

### 4.1. pH of Aqueous Solution

In many synthesis processes [28,56], the pH of the aqueous solution is considered the most important parameter influencing their efficiency, as its value affects: (i) the speciation of precious metal ions—the form of free ions or complex species, as well as the apparition or retrogradation of secondary equilibriums (such as: precipitation, hydrolysis, etc.); (ii) the degree of protonation or ionization of functional groups on the surface of the algae biomass, which are involved in redox processes; and (iii) the stabilization of the synthesized PM-NPs by favoring electrostatic repulsion interactions (see Section 3).

Precious metal ions are found in aqueous solution as simple chemical species (free ions or complex species) in acid media (pH = 0–6.0). In this form, they can readily participate in redox (reduction) processes and can generate nanoparticles, according to the processes:

Ag^+^ + e^−^ → Ag^0^ (2)

AuCl_4_^−^ + e^−^ → Au^0^ + 4 Cl^−^ (3)

Pt(Pd)Cl_4_^2−^ + 2e^−^ → Pt^0^(Pd^0^) + 4 Cl^−^ (4)

Speciation diagrams from the literature show that gold exists as AuCl_4_^−^ ions up to pH values of 6.5 [118], silver as free ions up to pH 6.0 [119], while for platinum and palladium, the tetrachloride complex forms are predominant up to pH values of 7.5 [120]. At higher pH values, precious metal ions can be involved in hydrolysis equilibriums and the resulting basic salts exhibit a much lower tendency to be reduced, which leads to a decrease in the efficiency of synthesis processes. Therefore, from this perspective, it is preferable for the pH of the aqueous solution to be as low as possible (acid media). Anyway, the presence of PM ions as complexes (such as gold (AuCl_4_^−^), platinum, and palladium (PtCl_4_^2−^ and PdCl_4_^2−^)) increases the pH value at which their precipitation begins, allowing their use for PM-NP synthesis even in basic media [109,114].

However, in the same aqueous solution (containing precious metal ions), algae biomass is also introduced. It is well known that, in acidic media, most of the superficial functional groups of algae biomass are either protonated (positively charged) or undissociated [75], which facilitates the development of redox processes (Figure 4). From this perspective, the pH value must be selected carefully, because at too-low pH values (strongly acidic media, favorable for the speciation of precious metal ions), some functional groups on the surface of the algae biomass may degrade or be involved in intra/inter particle hydrogen bonds [69,75], which significantly reduces their availability to participate in redox processes. This is much more important in the case of using algae extracts in the synthesis processes of PM-NPs, where the organic compounds in the extracts can degrade much more easily at low pH values compared to algae biomass [54]. 

Fortunately, the degradation of functional groups on the surface of algae biomass is most often minimized due to its buffering behavior. Studies in the literature [75,112] show that when algae biomass is introduced into acidic aqueous solutions (pH = 2.0–4.0), the final pH of the solution increases to 4.0–6.5 (depending on the type of algae). Such a rise in pH, from acidic to weakly acidic or neutral, is attributed to the large number and diversity of functional groups on the surface of the algal biomass, which can stabilize the pH of the aqueous solution. This resistance to pH variation can also be observed in algae extracts, especially in those obtained at high temperatures and extended extraction times, which are characterized by complex chemical compositions (a large and diverse number of extracted organic compounds) [75,121].

The pH value of the aqueous solution also influences the stability of PM-NPs in solution. A sufficiently high concentration of protons (acid media) can prevent the aggregation of the formed PM-NPs due to electrostatic repulsion interactions (see Figure 7). This can also be achieved by adding stabilizing agents, a method used when highly acidic aqueous media may affect the efficiency of PM-NP synthesis (see previous section). As a fairly general rule, acidic media (pH < 4.0) favor the formation of small PM-NPs, but with modest stability, while higher pH values (pH = 8–10) allow the synthesis of larger PM-NPs that are well stabilized [49,76]. 

Due to the multiple consequences that the pH variation can have on the development of elementary processes, the efficiency of PM-NP synthesis depends to a large extent on the value of this parameter. Therefore, the influence of pH on the efficiency of PM-NP synthesis processes must be experimentally examined for each case, and the optimal value of this parameter must be selected. Table 6 summarizes the experimental conditions used for the synthesis of PM-NPs in the case of some algae biomasses (or extracts). Unfortunately, in many of these examples, little attention has been paid to pH, with the focus primarily on the practical applicability of the obtained PM-NPs, even though their synthesis requires high temperatures or long contact times. 

### 4.2. Algae Biomass (Extract) Dosage

The dosage of algae biomass (or extract) is another parameter that significantly influences the efficiency of PM-NP synthesis. 

This is because the higher the amount of biomass (or extract), the higher the ratio between the number of functional groups of the biomass and the number of PM ions in the aqueous solution. This increase in the ratio between the number of functional groups and the number of PM ions leads to an increase in the efficiency of PM-NP synthesis, because [90,121] (i) the number of metal ions involved in the reduction process increases, and (ii) the time required to obtain PM-NPs decreases. 

**Figure 10:**
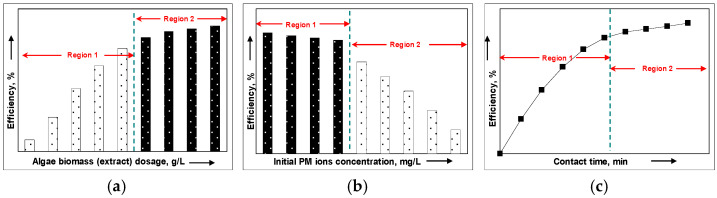
Illustrative representation of the influence of algae biomass dosage (**a**), initial PM ions concentration (**b**), and contact time (**c**) on the efficiency of PM-NP synthesis.

Unfortunately, the relationship between the amount of biomass and the efficiency of PM-NP synthesis is not linear. In the graphical representation of the synthesis efficiency as a function of biomass dosage (Figure 10a), two regions can be distinguished, regardless of the type of algae biomass or the nature of the PM ion in the aqueous solution. In the first region (Region 1), the synthesis efficiency increases significantly with the biomass dosage, indicating that the number of PM ions is still much higher compared to the number of functional groups available for their reduction. In the second region (Region 2), the efficiency of PM-NP synthesis increases much more slowly with the rise in algae biomass dosage, which suggests that the number of functional groups required for the reduction of PM ions is sufficient. 

Therefore, the value of this parameter must be optimized (most often through experimental tests) so that for a given synthesis process, it ensures high efficiency in obtaining PM-NPs without additionally increasing the synthesis costs (due to large amounts of algae biomass or higher energy consumption). Table 6 presents some examples of biomass dosages used in studies in the literature. 

### 4.3. Concentration of PM Ions in Aqueous Solution

For a given pH value and algae biomass dosage (established as optimal), increasing the initial concentration of PM ions in the aqueous solution leads to a decrease in the percentage of PM-NPs obtained through synthesis. A schematic representation of this variation is illustrated in Figure 10b. 

In general, the increase in the initial concentration of PM ions leads to a decrease in the efficiency of PM-NP synthesis. This decrease is determined by the decrease in the ratio between the number of functional groups on the surface of the algae biomass and the number of PM ions in the solution [94,127,131]. Thus, the higher the initial concentration of PM ions, the higher the ion density around the functional groups, which means that not all PM ions will find favorable conditions for their reduction. Consequently, the efficiency of the PM-NP synthesis process (which mathematically involves referencing the initial concentration of PM ions) decreases (Figure 10b). In this case, the delimitation of the two regions (1 and 2) is made according to the values of synthesis percents, but most often, Region 1 corresponds to the PM ions concentration range in which the synthesis of PM-NPs is quantitative (>90%), while Region 2 includes PM ions concentration values for which the synthesis efficiency is below 75% (not quantitative). 

The concentration range of PM ions corresponding to the two regions is established (experimentally) for each case, and depends on the nature of the PM ion, the type and amount of algae biomass, and the experimental conditions (pH, contact time, temperature, etc.) [132,134]. Therefore, depending on the proposed objectives (testing the efficiency of the synthesis process or obtaining PM-NPs with well-defined sizes), the experimental studies may use either a specific initial concentration value of PM ions or a concentration range. Several examples are summarized in Table 6. However, as a general rule, it is expected that for low initial concentrations of PM ions, the efficiency of the synthesis process will be high and the obtained PM-NPs will have smaller sizes and well-defined shapes. In contrast, at high initial concentrations of PM ions, the synthesis efficiency tends to decrease, and the obtained PM-NPs may exhibit a wider range of sizes and shapes [94,97].

### 4.4. Contact Time

Since the synthesis of PM-NPs using algae biomass (solid) largely depends on how easily the reduction of PM ions (aqueous solution) occurs, the contact time is another experimental parameter that can significantly influence the efficiency of this process. Thus, the longer the contact time between the two phases (solid and aqueous solution), the higher the efficiency of PM-NP synthesis. This is why, in many studies from the literature, the selected contact time for PM-NP synthesis is very long (e.g., 24 h or 1440 min), regardless of the nature of the PM ion or the algae biomass (Table 6). 

However, a high contact time also has several drawbacks that affect the practical applicability of PM-NP synthesis processes. The most important of these are [94,124]:

(i) High energy consumption—determined by the need to keep the two phases (algae biomass and PM ion solution) in contact (by stirring at a certain temperature) for a long time. This drawback can be minimized by using algae extracts instead of raw algae biomass, for which the required contact time is shorter (Table 6).

(ii) Changes in the shape and size of the synthesized PM-NPs—the longer the contact time between the two phases, the higher the probability of agglomeration of the synthesized PM-NPs. Consequently, at high values of the contact time, stable PM-NPs with larger sizes and varied shapes are obtained, while at low values of contact time, although the stability of the obtained PM-NPs is lower, their sizes and shapes are more well-defined [126,127]. 

(iii) The uselessness of high contact time values—determined by the fact that the dependence between the efficiency of PM-NP synthesis and contact time is not linear (Figure 10c). Under well-defined experimental conditions (a specific pH of the initial solution, a certain dose of algae biomass, and a given initial concentration of PM ions), the efficiency of the synthesis processes increases significantly at low contact time values (Region 1), after which equilibrium is reached and the increase in PM-NP synthesis efficiency becomes much slower (Region 2) [127]. The significant increase in PM-NP synthesis efficiency at low contact time values (Region 1—Figure 10c) is due to the large number of functional groups available for the reduction of PM ions. When the number of functional groups available to participate in the reduction of PM ions decreases (due to their oxidation), further increasing the contact time no longer brings significant improvements in the efficiency of the PM-NP synthesis (Region 2—Figure 10c). 

Therefore, the minimum contact time required to ensure efficient synthesis of PM-NPs must be experimentally evaluated, depending on the working conditions, the type of PM ion, as well as the characteristics (number and nature of functional groups) of the algae biomass (extract). In general, for an efficient PM-NP synthesis process, the minimum contact time represents the duration required to convert 90% of the initial concentration of PM ions into nanoparticles under given experimental conditions [94]. An additional 60–120 min may be added to this value to ensure that the synthesis process reaches equilibrium. Any further increase in contact time becomes unnecessary, as it only slightly improves the synthesis efficiency (by a few percent) but can significantly affect the size and shape of the obtained PM-NPs [94].

### 4.5. Temperature

Temperature is another experimental parameter that can influence the efficiency of PM-NP synthesis, and its effect is directly related to the variation in the kinetic energy of the ions and molecules involved in the synthesis processes [126,130]. In most cases, increasing the temperature within a certain range (10–100 °C) leads to an increase in thermal agitation in the synthesis solutions, and consequently to an increase in the efficiency of PM-NP synthesis. This increase in the efficiency of PM-NP synthesis with increasing temperature can be explained by considering the elementary processes involved in PM-NP synthesis (see Section 3). Thus, as the temperature increases [130]: (i) the elementary transport processes occur at a higher rate—the diffusion of PM ions to the surface of the algae biomass (or near the molecules in the algae extract) and the movement of the obtained PM-NP in the solution bulk are carried out more easily, and (ii) the electron transfer required for the reduction of PM ions takes place more quickly, which allow efficient and rapid obtaining of PM-NPs.

However, the temperature increase must be carried out within a well-defined range, mainly depending on the type of algae biomass (or extract) used in the synthesis process. This is because, in addition to increasing the efficiency of PM-NP synthesis, high temperatures can influence [94]:

(i) *The stability of the algae biomass (extract)*—due to unwanted secondary degradation processes that may occur at high temperatures and which can reduce the number of functional groups available for PM ion reduction, thereby directly affecting the efficiency of the synthesis process.

(ii) *The efficiency of stabilizing agents*—temperature variation influences the adsorption or complexation of stabilizing ions or molecules on the surface of the formed PM-NPs. Unfortunately, it is quite difficult to formulate a general rule, as the efficiency of stabilizing agents depends not only on their type and concentration but also on the nature of the PM-NPs and the experimental synthesis conditions.

(iii) *The physicochemical characteristics of the obtained PM-NPs*, such as [139]:

(a) PM-NP size—higher temperatures can accelerate the agglomeration of the nanoparticles, resulting in larger PM-NPs, while lower temperatures favor the formation of smaller nanoparticles;

(b) Shape and morphology—temperature influences surface energy and the mobility of ions/molecules in the aqueous solution, which can lead to the formation of PM-NPs with various shapes (spherical, star-like, cubic, etc.);

(c) Size distribution—lower temperatures often allow for the synthesis of PM-NPs with a relatively uniform size distribution, compared to higher temperatures, where the size distribution becomes non-uniform due to agglomeration;

(d) Optical and electronic properties—the temperature used in the synthesis processes of PM-NPs influences their electronic structure and optoelectronic behavior, allowing the obtaining of PM-NPs that have increased or, conversely, reduced conductive properties.

All these observations show that establishing the optimal temperature value is essential for the efficient synthesis of PM-NPs with well-defined characteristics. Table 6 presents some examples of optimal temperature values used in the synthesis of PM-NPs employing algae biomass (or extracts). 

Recent studies in the literature [80,90,94,97] recommend that, in advanced synthesis processes that aim to obtain PM-NPs with well-defined physicochemical characteristics (such as size, shape, physical properties, etc.), the mixture of algae biomass (extract) and PM ions solution should be successively exposed to high and then low temperatures during the synthesis process. However, this successive exposure to different temperature values must follow a rigorously developed protocol, and the temperature limits (maximum and minimum) must be well established in order to obtain the desired PM-NPs.

In addition to pH, algae biomass (extract) dosage, initial concentration of PM ions, contact time, and temperature, many other experimental factors can influence the efficiency of PM-NP synthesis. The type of algae biomass (extract) also plays an important role in the synthesis processes of PM-NPs, because different types of algae (extracts) contain in their composition different types of functional groups that may, or may not, participate in the reduction of PM ions from solution [140]. Therefore, the proper selection of algae biomass (extract) can significantly influence the efficiency of PM-NP synthesis. The stirring (mixing) speed of the algae biomass (extract) and the aqueous PM ion solution also influences the efficiency of the synthesis processes. The higher the stirring speed, the better the contact between PM ions and the functional groups of the algae biomass (extract), which leads to an increase in the efficiency of the reduction (redox) processes [120]. However, a high stirring speed also increases the probability of collisions between the formed PM-NPs, thereby favoring their agglomeration. Therefore, it is necessary to establish an optimal value for this parameter as well.

All these parameters must be experimentally optimized for each synthesis process in order to evaluate the efficiency of obtaining PM-NPs and to establish their practical applicability. Even when optimization programs (Surface Response Methodology, Experimental Design, Artificial Neural Networks, Genetic Algorithms, etc.) are used to reduce the number of experiments, the values indicated as optimal must be experimentally verified. The lack of optimal values for these parameters in many studies in the literature is the main gap in comparing different PM-NP synthesis processes, in terms of their efficiency.

## 5. Characterization Methods of PM-NPs

Comprehensive characterization of PM-NPs synthesized using algae biomass (extract) is essential for understanding their properties and establishing potential applications. However, due to the complexity of the synthesis processes, detailed characterization of the obtained PM-NPs can be time-consuming and expensive, due to the large number of analytical methods that must be used for this purpose [77,94]. Therefore, selecting relevant characterization methods that allow the identification of the most important characteristics of PM-NPs is another important aspect to consider.

In choosing the appropriate methods for the characterization of PM-NPs, the main selection criteria to be considered are: 

(i) Information regarding the structure of the nanoparticles; 

(ii) The working time and cost of the characterization method; 

(iii) The availability of analytical equipment. 

**Figure 11:**
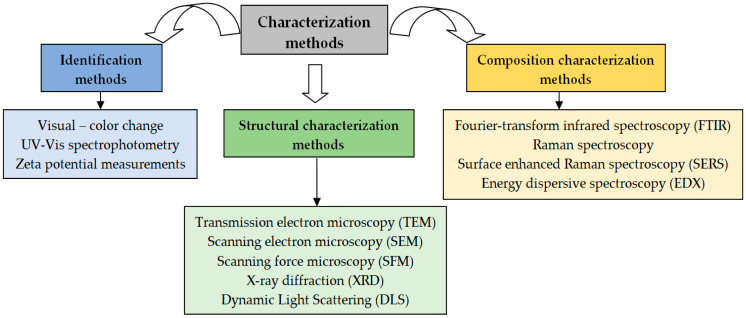
Classification of PM-NP characterization methods.

Based on these criteria, the characterization methods can be divided into three categories, schematically illustrated in Figure 11. 

### 5.1. Identification Methods

In general, identification methods are used only to obtain qualitative information that confirms the formation of PM-NPs in a given system, considered for synthesis. This information is essential when testing new synthesis processes, as it may or may not encourage their further examination.

#### 5.1.1. Visual—Color Change

**Figure 12:**
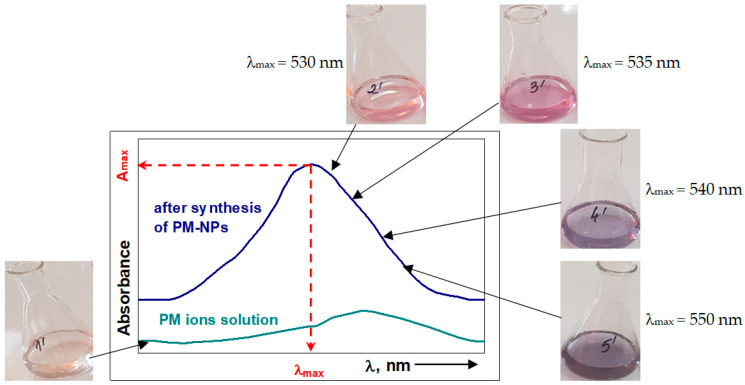
Schematic illustration of the color change and the shape change of the Vis spectrum for the synthesis of Au-NPs using *Ulva lactuca* algae biomass.

When examining a synthesis process, the formation of PM-NPs is most readily observed visually, due to the color change of the solution (Figure 12). The color change depends both on the nature of the PM ions in the solution and on the experimental conditions that influence their size, and therefore, the information obtained is strictly qualitative [141]. 

However, the visual identification of color changes is performed quickly, without additional costs and without altering the composition of the synthesis systems, and therefore can be considered the first condition to be fulfilled in the design of a PM-NP synthesis process.

#### 5.1.2. UV–Vis Spectrophotometry

UV–Vis molecular absorption spectrometry (UV–Vis spectrophotometry) is a method most often used to confirm the formation and stability of PM-NPs in a given synthesis process. This method involves recording molecular absorption spectra (A = f(λ, nm)) in the UV–Vis range (230–760 nm), and allows the determination of the concentration of PM-NPs in the solution (using the Lambert–Beer law) as well as their average size (based on the wavelength corresponding to the absorption maximum (λ_max_)). Due to the color change of the solution as a result of the formation of nanoparticles (Figure 12), this method can be used to identify most PM-NPs, which have sizes from 1 to 100 nm [142]. 

Unfortunately, the information obtained through this method (PM-NPs concentration and their size) is only semi-quantitative, due to the fact that absorbance (measured experimentally) is an additive parameter [52]. The presence of PM-NPs of different sizes (which have different colors) in the aqueous solution, or of other colored components (e.g., polyphenols from algae extracts), significantly affects the experimental values of absorbance and the position of the absorption maximum, and therefore the calculated values for the concentration and size of PM-NPs. To minimize experimental errors that may affect absorbance measurements in the solutions resulting from the synthesis of PM-NPs, the following aspects should be taken into consideration [143]: 

(i) The solutions used for absorbance measurements must be clear and homogeneous—solid particles (such as algae biomass or large PM-NPs aggregates) should be removed by filtration or centrifugation;

(ii) If the PM-NP synthesis solution contains colored compounds, the experimental measurement of absorbance should be performed against a blank sample that includes these compounds and not against distilled water;

(iii) The experimental measurements should be performed at intervals of 0.5 nm or less to accurately determine the position of the absorption maximum (λ_max_) and the absorbance value (A_max_), corresponding to this maximum.

UV–Vis spectrophotometry is a simple, rapid, inexpensive, and easy-to-use method, and therefore, recording UV–Vis spectra can be considered the next step in characterizing a given PM-NP synthesis process.

#### 5.1.3. Zeta Potential Measurements

PM-NPs obtained through synthesis using algae biomass can be characterized using zeta potential, which allows the evaluation of the colloidal stability of the formed nanoparticles and their behavior in aqueous solution [144]. As is well known, zeta potential is a measure of the electric potential at the surface of nanoparticles in a liquid suspension and reflects the interaction between the nanoparticle surface and the dissolved ions in the solution, which are necessary for the formation of the electric double layer [145]. 

Experimental measurement of zeta potential can be done using electrophoretic light scattering (ELS) or Laser Doppler micro-electrophoresis [142]. These methods involve applying an electric field to the solution containing PM-NPs, which causes them to migrate toward electrodes of opposite polarity. This movement of the PM-NPs is recorded and allows the calculation of electrophoretic mobility, and consequently, the zeta potential. Depending on the experimental zeta potential value, it can be established whether the PM-NPs obtained by synthesis, under the given conditions, are [145]:

(i) Stable and have a reduced tendency to agglomerate—characterized by high zeta potential values (above ±30 mV);

(ii) Unstable and have a pronounced tendency to agglomerate—characterized by low zeta potential values (between −10 mV and +10 mV);

(iii) Functionalized as a result of their interaction with other molecules in the solution.

The measurement of zeta potential is itself a simple, rapid, and non-destructive method that can be used in the characterization of PM-NPs. The information obtained through these measurements allows for the determination of PM-NPs size, but more importantly, their stability in aqueous solutions. While the accuracy of size determination is closely tied to the performance of laboratory equipment (which can be costly in certain cases), the evaluation of PM-NPs stability in solution is performed with reasonable precision, using this parameter. Therefore, zeta potential measurement can be considered the final step in evaluating the potential of a given PM-NP synthesis process.

All of these methods have been included in the category of identification techniques (see Figure 11) because they allow for the easy and rapid characterization of PM-NPs, even if the information obtained is of a semi-quantitative nature, and are most often used for optimization of synthesis processes. Once the optimal conditions ensuring high efficiency of the synthesis process have been established, PM-NPs must be characterized in much more detail, using the methods presented in the following sections. 

### 5.2. Structural Characterization Methods

In the category of structural characterization methods are included those techniques that provide important information about the size, shape, and crystalline structure of PM-NPs. The most important among these are scanning electron microscopy (SEM), transmission electron microscopy (TEM), scanning force microscopy (SFM), and X-ray diffraction (XRD) (Figure 11), and the key aspects related to their use in the characterization of PM-NPs are discussed below. 

#### 5.2.1. Scanning Electron Microscopy (SEM)

Scanning electron microscopy (SEM) is a non-destructive analytical method (an imaging technique) that can be used to examine the morphology of PM-NPs surfaces. From an analytical point of view, SEM involves [142,146]:

(a) The generation of an accelerated electron beam, which is directed to scan the sample surface point by point according to a grid pattern;

(b) The interaction between the accelerated electrons and the atoms on the sample surface, which generates different types of signals;

(c) The obtained signals are converted into a digital image to provide a detailed representation of the sample surface.

This results in high-resolution 3D images that allow for the visualization of fine surface details of PM-NPs, including possible aggregations or defects. 

Using this method, surface and dimensional measurements of PM-NPs can be performed, which allows the evaluation of the shape, size, and distribution of the nanoparticles. Spherical, cubic, dendritic, and other structures can be identified by SEM [146], details that are particularly useful for characterizing the synthesis process and the physical properties of the obtained PM-NPs. It is a fast, easy-to-use method that provides reliable details. 

Moreover, it can be easily coupled with other analytical techniques (e.g., energy dispersive X-ray spectroscopy (EDX)) to obtain information about the elemental composition and the concentration of specific elements in the analyzed samples [142]. Table 7 summarizes the most important advantages and disadvantages of using SEM in the characterization of PM-NPs. 

#### 5.2.2. Transmission Electron Microscopy (TEM)

Transmission electron microscopy (TEM) is one of the most advanced and precise techniques used in the characterization of PM-NPs. Due to its extremely high resolution, TEM enables direct observation of nanoparticles at the atomic scale, providing detailed information about their shape, size, and morphology [77,142]. These characteristics are essential for characterizing the synthesis process and assessing the practical applicability of the obtained PM-NPs. Unfortunately, such high-resolution images can only be obtained for PM-NPs with sizes ranging between 20 and 100 nm [147]. For nanoparticles larger than 100 nm, the resolution is reduced or only limited areas can be analyzed, while for nanoparticles smaller than 20 nm, images cannot be recorded. 

Similar to SEM, TEM allows the structural characterization (size, shape, distribution, etc.) of PM-NPs, with the amendment that the images obtained by TEM have a higher resolution than those obtained by SEM [147]. Unlike SEM, TEM offers the possibility to examine the crystalline structure of nanoparticles by electron diffraction, allowing the identification of the types of crystal lattice, the orientations of the atomic plane, and structural defects of the analyzed PM-NPs [77]. These details are particularly important for the evaluation of the structural properties and functional performance of PM-NPs. 

For a comprehensive characterization of PM-NPs, TEM can be coupled with energy dispersive X-ray spectroscopy (EDX), which enables the analysis of the elemental composition of the nanoparticles [142]. The most important advantages and disadvantages of this method are also presented in Table 7. 

#### 5.2.3. Scanning Force Microscopy (SFM)

Scanning force microscopy (SFM) is another important method in the characterization of PM-NPs, due to its ability to provide three-dimensional images (3D images) with nanometric resolution without destroying the analyzed samples. This method involves scanning the surface of the nanoparticles with an extremely sharp tip, which allows the determination of the size, shape, and distribution of individual or aggregated PM-NPs, with high precision [142]. Unlike TEM and SEM, SFM can analyze the surface of PM-NPs in various environments, including non-conductive ones and does not require vacuum conditions [148]. 

The high-resolution images obtained through SFM allow for the evaluation of the surface roughness of PM-NPs, as well as the measurement of mechanical properties (such as elasticity, adhesion, and hardness) of the nanoparticles [77]. These insights are highly relevant for determining their potential applications. 

Moreover, AFM can be coupled with other nanoparticle characterization techniques (such as Raman spectroscopy or FTIR spectroscopy), which allows the correlation of PM-NP topography with their chemical composition. The main advantages and limitations of this method are also summarized in Table 7.

#### 5.2.4. X-Ray Diffraction (XRD)

X-ray diffraction (XRD) is an essential method for the structural characterization of PM-NPs, as it allows the precise identification of the crystalline phase and provides information about their internal structure [142]. The crystalline structure describes the arrangement of atoms in the analyzed samples, and is revealed by the position and intensity of the diffraction peaks. Since the wavelength of X-rays is on the atomic scale, this method can be used to examine the structure of PM-NPs [149]. For example, Au-NPs and Ag-NPs have a face-centered cubic structure, which can be proved by XRD analysis [150]. Also, this method can detect possible impurities or secondary phases that may arise during the synthesis process, allowing real-time monitoring of the evolution of the crystalline structure and confirming the formation of the desired nanoparticles. 

Another major advantage of XRD is its ability to estimate the average crystallite size (using the Scherrer equation), a parameter that can be correlated with the size of the nanoparticles [149]. Moreover, XRD can assess the degree of crystallinity of the samples used for analysis and differentiate amorphous from crystalline materials. This information is particularly important, especially in the case of PM-NPs for which a low degree of crystallinity can significantly modify their practical utility (optical, electronic, or catalytic properties) [77,142]. 

However, XRD also has a number of limitations. It does not provide direct information about the shape or size distribution of PM-NPs, for which complementary techniques (such as TEM, SEM) are required. In addition, the sensitivity of the method decreases in the case of very small amounts of PM-NPs, or when the nanoparticles are extremely small (below 5 nm), where the XRD signal becomes weak or diffuse [142]. Nevertheless, XRD remains an indispensable method in the experimental strategy for the characterization of PM-NPs. 

#### 5.2.5. Dynamic Light Scattering (DLS)

Dynamic Light Scattering (DLS) is another method that can be used for the structural characterization of PM-NPs, allowing the determination of the hydrodynamic size of nanoparticles in suspension by analyzing their Brownian motion and the fluctuations in the intensity of scattered light [149]. In this way, rapid and accurate information is obtained about the average size of the nanoparticles, as well as the size distribution in the samples used for analysis [77]. Thus, using DLS, small volumes of suspensions (10–20 μL) containing PM-NPs with small sizes (ranging from 0.3 to 6.0 nm) can be analyzed, and the measurement accuracy is 2% [151]. 

Another major advantage of this method is that it offers the possibility of monitoring the colloidal stability of PM-NPs under various experimental conditions (such as pH, temperature, chemical composition, etc.), thus providing a clear picture of their aggregation or dispersion tendency [152]. These characteristics are important for assessing the reproducibility of synthesis processes and their practical applications.

Unlike the previously presented methods, the DLS method is considered a simple and rapid method that does not require complex sample preparation (Table 7). This method can be used directly on samples taken from the suspensions in which PM-NPs are synthesized, making it ideal for real-time monitoring of synthesis processes [151]. In addition to all these advantages, DLS also has a number of limitations (Table 7), which must be taken into account for the correct interpretation of the results obtained experimentally by this method.

However, in establishing practical applicability (such as in catalysis, plasmonic applications, or medical applications), where the size and stability of the synthesized PM-NPs are key factors, the use of DLS is recommended for nanoparticle characterization. 

All these structural characterization methods (Figure 11) provide valuable information about the size, shape, and crystalline structure of PM-NPs. Unfortunately, this information is not always sufficient to fully understand the nature of the interactions that may occur during the synthesis processes of PM-NPs using algae biomass (extract). Therefore, these methods must be complemented by compositional characterization methods, which will be presented in the next section.

### 5.3. Composition Characterization Methods

The characterization methods (Figure 11) included in this category (Fourier-transform infrared spectroscopy (FTIR), Raman spectroscopy, surface-enhanced Raman spectroscopy (SERS), and energy dispersive spectroscopy (EDX)) provide detailed information about the chemical composition of both PM-NPs and other components (organic molecules from algae biomass or extracts) involved in the synthesis processes. Moreover, the use of these methods in situ allows for obtaining particularly useful information about the elementary processes that occur during the formation and stabilization of PM-NPs.

#### 5.3.1. Fourier-Transform Infrared Spectroscopy (FTIR)

Fourier-transform infrared spectroscopy (FTIR) is a commonly used analytical method for the chemical characterization of PM-NPs. Although it does not provide atomic-level structural information, FTIR enables the identification of specific chemical bonds associated with functional groups present on the surface [142,153]. Thus, FTIR spectroscopy can reveal groups such as hydroxyl, carboxyl, carbonyl, amino, etc., which play a crucial role in the stabilization and reactivity of PM-NPs. These functional groups may originate from algae biomass (extracts), stabilizing agents, or molecules used for surface functionalization, and their presence significantly influences the physicochemical properties of PM-NPs [77]. 

This method is particularly useful for monitoring the synthesis process of PM-NPs using algae biomass (extract), as it allows the observation of chemical changes occurring during their synthesis. By comparing FTIR spectra obtained at different stages of the synthesis process, interactions between PM ions and the involved organic compounds can be highlighted, providing insights into the nucleation and growth mechanisms [153]. Moreover, this method can also be used to evaluate the efficiency of stabilizing agents by identifying the bonds formed between the surface of PM-NPs and their molecules [77]. 

Another major advantage of FTIR spectroscopy is its ability to study the behavior of PM-NPs in various environments, including biological or chemically aggressive ones. Spectral changes can indicate processes such as oxidation, aggregation, or degradation, thus being useful for assessing the stability and durability of PM-NPs in real applications [154]. Although FTIR spectroscopy does not allow the identification of the nature of PM ions in the PM-NPs composition, this method can be successfully used to complement the information obtained from other characterization methods (such as scanning electron microscopy (SEM), transmission electron microscopy (TEM), or X-ray diffraction (XRD)), contributing to a comprehensive and integrated understanding of the processes involved in PM-NP synthesis. The most important advantages and disadvantages of FTIR spectroscopy are summarized in Table 8.

#### 5.3.2. Raman Spectroscopy and Surface-Enhanced Raman Spectroscopy (SERS)

Raman spectroscopy can also be used in the characterization of PM-NPs to obtain detailed information about molecular structure, chemical interactions, and changes that occur during the synthesis or functionalization processes of PM-NPs [154]. Raman spectroscopy allows the analysis of vibrational modes of molecules adsorbed on the surface of nanoparticles and the identification of specific molecular “fingerprints”, thereby contributing to the understanding of the elementary steps involved in the synthesis processes of PM-NPs [155].

But, Raman spectroscopy has a moderate sensitivity and is susceptible to interferences caused by fluorescence phenomena [155]. To minimize these disadvantages, the SERS (surface-enhanced Raman spectroscopy) variant was developed, which amplifies the Raman signal and allows the detection of extremely low concentrations of molecules (in the ppm or even ppb range) [142]. The SERS variant of Raman spectroscopy is ideal for the characterization of PM-NPs that require functionalization and are designed for medical applications. However, the use of the SERS variant of Raman spectroscopy is quite limited due to the complex methodology required for sample preparation, which involves special substrates (highly roughened or nanostructured metallic surfaces), making the cost of such analysis relatively high [154]. 

Unlike FTIR spectroscopy, which allows the identification of only those bonds that have a permanent or induced dipole moment (polar functional groups), Raman spectroscopy can be used to identify non-polar functional groups (bonds without a permanent electric dipole), which are, however, compatible with aqueous media [155]. Together, these two spectrometric methods (FTIR and Raman) provide a comprehensive picture of the nature of functional groups and how they are involved in the synthesis and/or functionalization of PM-NPs.

#### 5.3.3. Energy Dispersive X-Ray Spectroscopy (EDX)

Energy dispersive X-ray spectroscopy (EDX) is a method that allows the precise identification of the elemental composition of the analyzed samples (Table 8). This allows the presence of a specific precious metal to be confirmed, and any impurities or contaminants to be detected [142,156], which is crucial in the characterization of synthesized PM-NPs. 

In addition to element identification, EDX also provides quantitative or semi-quantitative information about the content of each element [156]. This aspect is particularly useful in determining the purity of PM-NPs and in monitoring the efficiency of synthesis processes. Moreover, when used in combination with scanning electron microscopy (SEM), this method allows the creation of elemental distribution maps on the surface of the analyzed samples. These visual maps help to understand how precious metals are distributed in the synthesized PM-NPs, whether they are uniformly distributed or if there are aggregation tendencies [123]. 

Nevertheless, EDX also has a number of limitations (Table 8). The spatial resolution is not as high as in the case of other techniques (e.g., TEM), and the detection of light elements (with atomic numbers lower than 13) is difficult [156]. However, in the context of the characterization of PM-NPs, EDX remains an indispensable tool, offering a valuable combination of precision, rapidity, and analytical versatility. Some of the most important advantages and disadvantages of this method are also presented in Table 8. 

In addition to the methods presented in this section, many other analytical techniques can be used for the characterization of PM-NPs synthesized using algae biomass. Thus, depending on the equipment available in the analytical laboratory and the specific parameters required for characterization, methods such as atomic force microscopy (AFM), energy dispersive X-ray absorption spectroscopy (EXAFS), electrophoretic light scattering (ELS), and many others can be successfully used to determine the structural and compositional characteristics of PM-NPs. Regardless of the selected methods, it is recommended that, for a comprehensive characterization of PM-NPs, an experimental strategy be designed that includes two (or more) methods from the three categories illustrated in Figure 11. Table 9 presents several examples of such combinations of characterization methods used in the case of PM-NPs. 

## 6. Challenges and Future Research

Studies in the literature have shown that the use of algae biomass for the synthesis of PM-NPs is a sustainable and efficient method, which allows for obtaining a wide variety of nanoparticles, with varied shapes, morphologies, and properties. Moreover, due to the use of algae biomass, this synthesis method is cost-effective and can be classified as an environmentally friendly (green) approach. Moreover, due to their significant applications in medicine, optics, electronics, and other strategic industrial activities, the market demand for PM-NPs is growing exponentially.

However, the large-scale production and commercialization of PM-NPs synthesized using algae biomass is still quite limited, which in turn restricts the development of new practical applications or the reduction in costs for existing ones. These limitations are mainly due to the following challenges, for which solutions are still being sought:

(1) The lack of detailed and comparative studies regarding the efficiency of PM-NP synthesis processes, which would allow the selection of the appropriate algae biomass and optimal experimental conditions (such as pH, PM ions concentration, biomass dosage, contact time, temperature, etc.). The absence of these working details makes it difficult to design a synthesis strategy capable of obtaining PM-NPs with uniform morphology and a narrow particle size distribution, as required by industrial applications.

(2) The development of a standardized experimental methodology that allows obtaining PM-NPs with closely similar structural characteristics under identical experimental conditions, regardless of the laboratory in which they are synthesized. Such a methodology should also take into account the preparation protocol of the algae biomass, since its composition plays a crucial role in determining the shape, morphology, and synthesis efficiency of PM-NPs.

(3) Elucidating the synthesis mechanisms of PM-NPs using algae biomass is another challenge that needs to be overcome to enhance the industrial applicability of PM-NPs. Identifying the experimental conditions and the key components in the algae biomass that are directly involved in the redox processes of PM ions will allow streamlining the synthesis procedures and a more accurate assessment of the implementation costs. 

(4) The separation, purification, and stabilization of PM-NPs synthesized using algae biomass are other aspects that require clarification and solid scientific grounding. Therefore, detailed experimental protocols are needed to outline the necessary steps for obtaining PM-NPs with a specific degree of purity (required by large-scale applications), as well as a clear specification of the time period during which these nanoparticles remain stable (in terms of shape, size, and morphology).

(5) The scalability of PM-NP synthesis processes using algae biomass from a laboratory level to an industrial scale can be considered another major challenge. The lack of experimental studies that translate all the experimental details required for such synthesis processes to large-scale makes, at least for now, difficult to achieve a PM-NPs production that meets the huge market demands.

(6) In addition to all these technical challenges, there is a pressing need for a rigorous toxicological assessment of the PM-NPs obtained through these synthesis processes, which would allow for the alignment of economic and environmental policies, thereby facilitating the potential international trade of PM-NPs. 

Therefore, future research in this field should focus not only on overcoming these challenges but also on identifying new and more elaborate applications of PM-NPs that can enhance the quality of human life. 

## 7. Conclusions

Due to their unique characteristics, PM-NPs have numerous applications in various fields (such as catalysis, electronics, optics, medicine, etc.), which has led to a significant increase in market demand for such nanoparticles, and an open and challenging research direction. Therefore, it is necessary to develop efficient synthesis methods for PM-NPs that are environmentally friendly and easy to implement on a large scale. In this context, the use of algae biomass for the green synthesis of PM-NPs can be a viable alternative, as algae are available in many regions of the world, are easy to cultivate, and have a rapid growth rate. This study aims to provide a comprehensive overview of how PM-NPs can be synthesized using algae biomass. The importance of experimental conditions (such as pH, contact time, biomass quantity, type of algae biomass, etc.) on the efficiency of the synthesis processes, as well as the elementary steps involved in obtaining PM-NPs, is discussed in detail in this study. Special attention has also been paid to the characterization methods of PM-NPs obtained through such synthesis processes, which can provide the necessary evidence for their structural and compositional description. These characteristics (size, shape, morphology, composition, etc.) are particularly useful in establishing the possible practical applications of the synthesized PM-NPs. Addressing the current challenges in this field and overcoming practical difficulties could open new perspectives in which the synthesis of PM-NPs using algae biomass can be applied not only at the laboratory scale, but also at the industrial level. 

## Figures and Tables

**Table 1:** Structural characteristics of considered precious metals [52,69].

Precious Metal	Au	Ag	Pt	Pd
Atomic number (Z)	79	47	78	46
Atomic weight (A)	196.97	107.87	195.08	106.42
Oxidation state	**+3**, +1	**+1**	**+2**, +4	**+2**, +4
Electronegativity (Pauling scale)	2.54	1.93	2.28	2.20
Standard redox potential, V	+1.5000	+0.7994	+1.2000	+0.9200
Density (at 20 °C), g/cm^3^	19.283	10.503	21.452	12.007
Meting point, °C	1064.18	961.78	1768.3	1554.9
Atomic radius, pm	144.0	144.0	139.0	137.0
Ionic radius, pm	85.0	126.0	62.5	64.0

**Table 2:** Stability constant of chloride complexes of precious metal ions [52].

Precious Metal	Chemical Reaction	log β
Au	Au^3+^ (H^+^) + 4 Cl^−^ → AuCl_4_^−^	24.49
*Ag*	*Ag*^+^ (*H*^+^) + *Cl*^−^ → *AgCl* ↓	*1.1 × 10*^−10^ *
Pt	Pt^2+^ (H^+^) + 4 Cl^−^ → PtCl_4_^2−^	13.99
Pd	Pd^2+^ (H^+^) + 4 Cl^−^ → PdCl_4_^2−^	27.20

(*) value of the solubility product.

**Table 3:** Marine algae biomass with demonstrated efficiency in the synthesis of PM-NPs.

Marine Algae	Type of Algae	PM-NPs	Notable Features	Reference
*Ulva lactuca*	Green algae	Au, Ag	Successfully used in eco-friendly synthesis	[84]
*Caulerpa racemose*	Green algae	Ag	Spherical and triangle stable nanoparticles	[85]
*Cladophora vagabunda*	Green Algae	Au	High efficiency, long stable nanoparticles	[86]
*Kappaphycus alvarezii*	Red algae	Au, Ag	Stable and monodisperse nanoparticles	[87,88]
*Gracilaria edulis*	Red algae	Au	Produces uniform and stable nanoparticles	[89]
*Galaxaura elongata*	Red algae	Au	High efficiency, stable and spherical nanoparticles	[12]
*Portieria hornemannii*	Red algae	Ag	Stable and monodisperse nanoparticles	[90]
*Halymenia dilatata*	Red algae	Pt	Stable and monodisperse nanoparticles	[91]
*Fucus vesiculosus*	Brown algae	Au	Active functional groups (carboxyl, phenol); increased efficiency	[80]
*Padina pavonica*	Brown algae	Au	Efficient and rapid synthesis	[92]
*Padina gymnospora*	Brown algae	Pt	Efficient and rapid synthesis	[93]

**Table 4:** Experimental conditions for the preparation of extracts from algae biomass.

Algae	g, Biomass	Solvent	T, °C	t, min	Reference
*Laurencia papillosa*	5.0	distilled water	70–80	5	[96]
*Ulva lactuca*	1.0	distilled water	70–80	45	[97]
*Halopteris scoparia*					
*Ulva rigida*	10	distilled water	70	15	[98]
*Gracilaria foliifera*					
*Cystoseira myrica*					
*Undaria pinnatifida*	15.0	1.5 mol/L ethanol	microwave	200	[99]
*Sargassum fusiform*					
*Undaria pinnatifida*		distilled water	100	15	[100]
*Pterocladia capillacea*	5.0	distilled water	ultrasonication	240	[101]
*Champia parvula*	1.0	distilled water	60	20	[102]
*Bostrychia tenella*	100	methanol	Room temperature	-	[103]
*Laurencia obtusa*					
*Cystoseria* sp.	2.0	distilled water	60	20	[104]
*Spatoglossum asperum*	5.0	distilled water	60	20	[105]
*Saragassum cervicorne*	5.0	distilled water	85–90	60	[106]

**Table 5:** Some examples of organic stabilizing agents used in the synthesis of PM-NPs.

PM-NPs	Stabilizing Agent	Size, nm	Shape	Reference
Au	Sodium citrate	3.5–4.0	Spherical	[107]
	Poly-vinyl-pyrrolidone	37.0 ± 2.0	Nano-stars	[109]
	Cetyltrimethylammonium bromide	144.0 ± 25.0	Nano-prisms	[110]
Ag	Sodium citrate	10.0–200.0	Spherical	[111]
	Citric acid	cca. 300	Nano-stars	[112]
	Poly-vinyl-pyrrolidone	15.0–35.0	Cubic	[113]
Pt	Poly-ethylen-imine	4.9	Spherical	[114]
		5.2	Cubic	
	Poly-vinyl-pyrrolidone	3.0–30.0	Octahedral	[115]
Pd	Poly-vinyl-pyrrolidone	5.0–15.0	Spherical	[116]
	Phosphonic acids	50.0	Nanodendrites	[117]

**Table 6:** Optimal experimental conditions for the synthesis of PM-NPs using algae biomass (extracts).

PM-NPs	Algae Biomass	pH	Biomass Dose	PM Ions Concentration, mg/L	Contact Time, min	Temperature, °C	Reference
Au	*Ulva lactuca*	2.0	4.0 g/L	40.0	1440	22	[86]
	*Cladophora vagabunda*	2.0	4.0 g/L	40.0	1440	22	
	*Callithamnion corymbosum*	4.0	4.0 g/L	240.0	60	21	[121]
	*Halymenia pseudoforesii*	-	1.0 g/L	150.0	20	60	[122]
	*Fucus vesiculosus*	7.0	1.0 g/L	100.0	480	23	[80]
	*Ecklonia cava* (extract)	-	1.0 g/mL	50.0	10	80	[123]
	*Sargassum wightii*	-	1.0 g/L	200.0	720	25	[124]
	*Sargassum muticum*	-	1.0 g/L	200.0	15	76	[125]
	*Undaria pinnatifida* (extract)	-	1.0 g/mL	50.0	1440	100	[94]
	*Turbinaria conoides*	2.0	2.0 g/L	100.0	60	-	[126]
	*Sargassum muticum*	2.6–3.2	4.0 g/L	50.0	75	-	[127]
Ag	*Ulva lactuca* (extract)	11.0	10 mg/mL	150.0	60	25	[128]
	*Ulva lactuca*	3.0	-	50–100	60	25	[97]
	*Caulerpa serrulata*	4.1	1.0 g/L	150.0	1440	27	[129]
	*Ulva armoricana*	3.0	0.5 g/L	100.0	360	20	[130]
	*Portieria hornemannii* (extract)	-	5.0 mL	150.0	1440	25	[90]
	*Sargassum muticum* (extract)	5.6	1.0 g/mL	55.0	30	35	[131]
	*Undaria pinnatifida* (extract)	-	0.5 g/mL	25.0	1440	100	[94]
	*Saccharina japonica* (extract)	-	50.0 mL	150–170	45	40	[132]
	*Sargassum* spp. (extract)	-	25.0 mL	25.0	60	80	[133]
	*Sargassum wightti*	-	1.0 g/L	150.0	1440	25	[134]
Pt	*Padina gymnospora* (extract)	-	10.0 mL	100.0	1440	100	[93]
	*Codium sp*. (extract)	-	10.0 mL	200.0	120	45	[135]
	*Ulva* sp.	7.8–8.0	3.0 g/L	0.1	720	20	[136]
Pd	*Codium* sp. (extract)	-	10.0 mL	100.0	120	45	[135]
	*Dictyota indica* (extract)	8.0	20.0 mL	120.0	120	60	[137]
	*Padina boryana* (extract)	-	5.0 mL	120.0	120	60	[138]

**Table 7:** The utility of structural methods for the characterization of PM-NPs.

Method	Advantages	Disadvantages	Reference
SEM	- evaluation of morphology, surface distribution - fast analysis - 3D images of the surface	- moderate resolution - required conductive coating	[146]
TEM	- evaluation of size, shape, and internal structure - very high resolution - detailed images	- high cost - complex sample preparation	[147]
SFM	- nanoscale topography - does not require vacuum - can analyze sample in liquid media	- longer analysis time - small scan area	[142]
XRD	- identification of structure and degree of crystallinity	- does not detect amorphous PM-NPs - requires sufficient amount of sample	[148]
DLS	- evaluation of the average size and the size distribution - short working time, simplicity - non-invasive method -allows direct analysis of suspensions	- high sensitivity to impurities - difficulties in analyzing polydisperse samples - does not provide morphological details	[149]

**Table 8:** The utility of the methods used for the composition characterization of PM-NPs.

Method	Advantages	Disadvantages	Reference
FTIR	- rapid and easy to use method - identification of functional groups - identifies surface modifications	- limited sensitivity for small PM-NPs	[153]
Raman	- reduced cost and complexity in sample preparation - non-destructive method, complementary to FTIR	- moderate sensitivity - possible interferences	[155]
SERS	- very high sensitivity - reduced interferences - destructive method	- high cost and complexity in sample preparation	[154]
EDX	- allows the determination of elemental composition - short working time - non-destructive method -integrable with SEM/TEM	- high sensitivity to impurities - low sensitivity to light elements	[156]

**Table 9:** Combinations of analytical methods used for the characterization of PM-NPs synthesized using algae biomass.

PM	Algae Biomass	Characterization Method	Size, nm	Morphology	Reference
Au	*Sargassum* sp.	UV–Vis, AFM, TEM, XRD, FTIR	300–400	hexagonal, truncated triangular	[125]
	*Laminaria japonica*	UV–Vis, TEM, XRD, FTIR	15–20	spherical	[157]
	*Fucus vesiculosus*	XRD, SEM, EDS, TEM, FTIR	20–50	spherical	[80]
	*Padina gymnospora*	UV–Vis, XRD, AFM, TEM, FTIR	8–21	spherical	[158]
	*Dictyota bartayresiana*	UV–Vis, FTIR, SEM	poly-size	spherical	[159]
	*Sargassum tenerrimum*	UV–Vis, Zeta potential, TEM, FTIR, DLS	5–45	polymorphic	[160]
	*Chondrus crispus*	UV–Vis, TEM, SEM, EDX, FTIR	30–50	spherical, polyhedral	[161]
	*Galaxaura elongata*	Zeta potential, TEM, FTIR	3.85–77.13	triangular, hexagonal	[12]
	*Ecklonia cava*	UV–Vis, XRD, SEM, TEM, FTIR, EDX	20–50	spherical, triangular	[123]
Ag	*Ulva reticulata*	UV–Vis, FTIR, SEM, XRD	40–50	spherical	[162]
	*Ulva lactuca*	UV–Vis, Zeta potential, FTIR, SEM, XRD	48.9	spherical	[84]
	*Ulva flexousa*	UV–Vis, XRD, FTIR, TEM	2–32	spherical	[163]
	*Pithophora oedogonia*	UV–Vis, EDX, SEM, DLS, FTIR	25–44	cubical, hexagonal	[164]
	*Spirogyra* sp.	UV–Vis, FTIR, TEM	40–80	spherical	[165]
	*Caulerpa serrulata*	UV–Vis, FTIR, XRD, TEM	10 ± 2	spherical	[129]
	*Caulerpa racemosa*	UV–Vis, XRD, TEM, FTIR	5–25	face-centered cubic	[166]
	*Gracilaria birdiae*	UV–Vis, Zeta potential, TEM, FTIR, DLS	20.30	spherical	[167]
	*Sargassum vulgare*	TEM, XRD, TEM, FTIR, EDX	10.00	spherical	[168]
Pt	*Padina gymnospora*	UV–Vis, XRD, SEM, TEM, EDX	5–50	octahedral	[93]
	*Caulerpa sertularioide*	UV–Vis, XRD, SEM, TEM, DLS, FTIR, EDX	6–22	spherical	[169]
	*Codium* sp.	UV–Vis, SEM, TEM, FTIR, EDX	15.97	cubic	[135]
Pd	*Sargassum ilicifolium*	UV–Vis, SEM	60–80	spherical	[170]
	*Sargassum bovinum*	UV–Vis, TEM, XRD, EDX, FTIR	5–10	octahedral	[171]
	*Codium* sp.	UV–Vis, SEM, TEM, FTIR, EDX	11.38	hexagonal	[135]

## Data Availability

The original contributions presented in the study are included in the article; further inquiries can be directed to the corresponding author.
